# Osimertinib as Neoadjuvant Therapy for Resectable Non-Small Cell Lung Cancer: A Case Series

**DOI:** 10.3389/fphar.2022.912153

**Published:** 2022-04-28

**Authors:** Yan Hu, Siying Ren, Lulu Yang, Zhongyi Tong, Ruoyao Wang, Wei Han, Chao Zeng, Jina Li, Peng Xiao, Li Wang, Fenglei Yu, Wenliang Liu

**Affiliations:** ^1^ Department of Thoracic Surgery, The Second Xiangya Hospital of Central South University, Changsha, China; ^2^ Hunan Key Laboratory of Early Diagnosis and Precision Treatment of Lung Cancer, The Second Xiangya Hospital of Central South University, Changsha, China; ^3^ Department of Respiratory and Critical Care Medicine, The Second Xiangya Hospital of Central South University, Changsha, China; ^4^ Research Unit of Respiratory Disease, Central South University, Changsha, China; ^5^ Hunan Diagnosis and Treatment Center of Respiratory Disease, Changsha, China; ^6^ Department of Pathology, The Second Xiangya Hospital of Central South University, Changsha, China; ^7^ Department of Cardiothoracic Surgery, The Third Xiangya Hospital of Central South University, Changsha, China

**Keywords:** neoadjuvant osimertinib therapy, epidermal growth factor receptor, objective response rate, pathologic downstaging, non-small cell lung cancer

## Abstract

**Background:** Evidence of osimertinib as neoadjuvant therapy for resectable non-small cell lung cancer (NSCLC) are currently lacking. This case series study aimed to assess the safety and feasibility of neoadjuvant osimertinib therapy followed by surgery for resectable NSCLC.

**Materials and methods:** Patients with resectable NSCLC with epidermal growth factor receptor (EGFR) mutation who received osimertinib as neoadjuvant therapy followed by surgery at our center were included. Demographic features, radiologic and pathological assessment of response, surgery-related details and complications, toxicity profiles, and prognostic outcomes were extracted.

**Results:** A total of 13 patients were included in this study. The median age at the time of surgical resection was 57 years (interquartile range: 52–64 years), and eight (61.5%) patients were female. The objective response rate (ORR) was 69.2% (9/13), and the complete resection rate was 100%. The rates of pathologic downstaging and lymph node downstaging were 100% (13/13) and 66.7% (6/9), respectively. There were no perioperative deaths and only three (23.1%) patients had postoperative complications. Seven (53.8%) and 13 (100%) patients experienced grade 1 treatment-related adverse reactions and laboratory abnormalities, respectively. No patients experienced drug withdrawal or surgical delays due to the adverse events. No patients showed grade 2 or worse toxicity profiles. One patient was lost to follow-up. The other 12 patients were alive and free of disease recurrence with a median follow-up time of 9.5 months.

**Conclusion:** Neoadjuvant osimertinib therapy seemed to be safe and feasible for resectable EGFR-mutated NSCLC. Future large prospective studies are warranted to confirm whether osimertinib as neoadjuvant therapy outperforms standard tyrosine kinase inhibitors (TKIs) or chemotherapy for resectable EGFR-mutated NSCLC.

## Introduction

Lung cancer is the second most commonly diagnosed cancer and the leading cause of cancer death worldwide (Sung et al., 2021). Non-small cell lung cancer (NSCLC) accounts for more than 85% of all lung cancers, of which 30%–40% are resectable cancers (Goldstraw et al., 2016). Radical surgical resection is the cornerstone of standard treatment for resectable NSCLC. However, approximately 25%–70% of patients with resectable NSCLC will eventually suffer relapse after complete resection (Duma et al., 2019). Even with postoperative adjuvant therapy for appropriate patients, the 5-year overall survival (OS) rate for patients with stage II-IIIa resectable NSCLC was only 41%–65% (Goldstraw et al., 2016).

Neoadjuvant therapy of resectable NSCLC is an emerging area of research. Neoadjuvant chemotherapy, aiming at increasing R0 resection rate and improving prognosis, showed an absolute 5% survival benefit at 5 years for patients with resectable NSCLC when compared with surgery alone (Group, 2014). However, there was no difference in overall and disease-free survival between preoperative and postoperative chemotherapy (Lim et al., 2009).

The advent of tyrosine kinase inhibitors (TKIs) has revolutionized the treatment of NSCLC harboring the mutation of epidermal growth factor receptor (EGFR). EGFR mutation, one of the most common genetic events in NSCLC, accounts for 17%–61% of lung adenocarcinoma cases reported in a series of studies, with most frequently detected in Asian, female, non-smoking patients (Kris et al., 2014; Kawaguchi et al., 2016). In-frame deletions around the LREA amino acid motif positions 747–750 in exon 19 and the L858R point mutation in exon 21 are the most common mutations, accounting for 85%–90% of all EFGR mutations together (Aoki et al., 2018). A series of studies have found that first- and second-generation TKIs have achieved improved progression-free survival (PFS) than platinum-based doublet chemotherapy in EGFR-mutated advanced lung cancer (Sequist et al., 2013; Wu et al., 2015). Osimertinib, a third-generation TKIs that originally invented to treat T790M-regulated resistance to first-generation TKIs, showed longer disease-free survival compared with placebo in patients with completely resected stage Ib-IIIa EGFR-mutated NSCLC as adjuvant therapy (Wu et al., 2020) and longer PFS and OS and a reduced risk of CNS metastases compared with first-generation TKIs for the treatment of EGFR-mutated advanced NSCLC (Ramalingam et al., 2020). A few scholars have currently explored the safety and efficacy of first/second generation TKIs as neoadjuvant therapy in resectable NSCLC. However, evidence of osimertinib as neoadjuvant therapy for resectable non-small cell lung cancer are lacking.

This study aimed to investigate the safety and feasibility of neoadjuvant osimertinib therapy followed by surgery for resectable NSCLC.

## Materials and Methods

### Patients

We retrospectively reviewed the clinical records of all patients with clinical stage Ib-IIIb resectable NSCLC with EGFR mutation who received osimertinib as neoadjuvant therapy followed by radical surgical resection at our center from July 2019 to October 2021. Preoperative staging workup included pretreatment tumor biopsy, contrasted-enhanced computed tomography (CT) scan of the chest, positron emission tomography (PET)/CT scan, brain magnetic resonance imaging (MRI), and invasive mediastinal nodal staging with endobronchial ultrasound (EBUS), as indicated. Tumors were staged based on the eighth edition of the lung cancer TNM staging system. The Clinical Research Ethics Committee of the Second Xiangya Hospital of Central South University approved this study (LYF2021096).

### Data Collection and Evaluation

The following clinical data were extracted, including demographic features, radiologic and pathological assessment of response, surgery-related details and complications, toxicity profiles, and prognostic outcomes. Comorbidity evaluation was determined by using Charlson Comorbidity Index (CCI) (Singh et al., 2016). Radiologic response assessment was determined according to Response Evaluation Criteria in Solid Tumors. The radiologic responses were classified as complete response (CR, no residual disease), partial response (PR, no less than 30% reduced in size), progressive disease (PD, no less than 20% increased in size or the occurrence of new lesions), and stable disease (SD, less than 20% increased and less than 30% reduced in size). Pathologic complete response (pCR) and major pathologic response (MPR) were considered as 0% and ≤10% of viable tumor cells remaining in residual tumor, respectively. Surgical information included extent of resection, surgical approach, dissected nodal station and number, operative time, estimated blood loss, postoperative hospital stay, chest tube duration, and surgical complications. Surgical complications were evaluated using the Society of Thoracic Surgeons database criteria. Toxicity profiles, including treatment-related adverse events (AEs) and abnormal laboratory findings, were graded using the National Cancer Institute Common Terminology Criteria for Adverse Events, version 5.0.

### Follow-Up

Follow-up was performed through outpatient visits or telephone calls. The final follow-up visit was set at March 2022.

### Statistical Analysis

Descriptive analyses of patient demographics, radiologic and pathologic evaluation of tumor response, surgical information, and toxicity profiles, and follow-up results were performed. Continuous variables were expressed as median and interquartile range (IQR). Categorical variables were expressed as numbers and percentages. All statistical analyses were conducted with STATA software.

## Results

### Patient Characteristics

From July 2019 to October 2021, 13 NSCLC patients underwent neoadjuvant osimertinib therapy followed by radical surgical resection. The demographic characteristics of these 13 patients are shown in Table 1. The median age at the time of surgical resection was 57 years (IQR: 52–64 years). There were eight (61.5%) female patients and five male patients. Of these 13 patients, three (23.1%) had a history of smoking. According to the CCI criteria for assessing comorbidities, 11 (84.6%) patients had a CCI score of 0, while one had a CCI score of 1 and 2, respectively. The preoperative clinical staging was as follows: one (7.7%) patient had stage Ib disease, six (46.2%) stage IIb, four (30.8%) stage IIIa and two (15.4%) stage IIIb. Preoperative NGS testing of the biopsy specimens showed the presence of 19del mutation in five (38.5%) patients, L858R mutation in six (46.1%), and L861Q mutation in two (15.4%).

**TABLE 1:** Patient characteristics.

Patient	Age	Sex	BMI	CCI	PS	%FEV1	%DLCO	cTNM	ypTNM	RR^a^	EGFR mutation
Case 1	71	Male	13.67	0	1	89	59	IIIb,cT3N2M0	IIIa,ypT1bN2M0	PD	L858R
Case 2	62	Female	21.72	0	1	89	116	IIb,cT3N0M0	Ia3,ypT1cN0M0	PR	L858R
Case 3	64	Female	23.61	0	1	94	92	IIIa,cT2bN2M0	Ia3,ypT1cN0M0	PR	L861Q
Case 4	55	Male	23.23	0	0	123	96	IIb,cT1cN1M0	Ia2,ypT1bN0M0	SD	L858R
Case 5	68	Female	23.49	0	0	113	103	IIb,cT2aN1M0	Ib,ypT2aN0M0	PR	L861Q
Case 6	56	Male	23.94	0	0	129	NA	IIIa,cT1bN2M0	Ia1,ypT1aN0M0	SD	19del
Case 7	46	Male	26.03	0	1	116	NA	IIb,cT3N0M0	Ia3,ypT1cN0M0	PR	19del
Case 8	52	Female	21.64	0	1	109	NA	IIb,cT2bN1M0	Ia2,ypT1bN0M0	PR	19del
Case 9	45	Female	21.48	0	1	99	NA	IIIa,cT4N0M0	IIa,ypT2bN0M0	PR	L858R
Case 10	67	Female	17.09	0	1	107	129	Ib,cT2aN0M0	Ia3,ypT1cN0M0	PR	L858R
Case 11	57	Female	24.62	2	1	76	89	IIIa,cT3N1M0	IIb,ypT1cN1M0	PR	L858R
Case 12	58	Male	21.08	1	0	100	NA	IIIa,cT2aN2M0	Ia3,ypT1cN0M0	SD	19del
Case 13	52	Female	24.84	0	1	95	81	IIIb,cT3N2M0	IIIa,ypT1bN2M0	PR	19del

a RR, radiologic response.

### Treatment Regimens and Response Assessment

The treatment regimens for all patients were determined jointly by the treating surgeons and oncologists in a multidisciplinary discussion mode. Osimertinib was given at a dosage of 80 mg QD for all patients and the median dosing time was 75 days (IQR: 60–90 days, Table 2). Preoperative CT evaluation showed PR in nine patients, PD in one and SD in three, with an objective response rate (ORR) of 69.2%. Postoperative pathologic evaluation showed pathologic downstaging in all of the 13 (100%) patients and lymph node downstaging in 6 (66.7%) of 9 pN1-3 patients. MPR or pCR was not observed in any patient.

**TABLE 2:** Treatment regimens and response assessment.

Duration of neoadjuvant therapy, days, median (IQR)	75 (60–90)
Radiologic response assessment	
PR	9 (69.2)
PD	1 (7.7)
SD	3 (23.1)
ORR	69.20%
Pathologic stage	
Ia1	1 (7.7)
Ia2	2 (15.4)
Ia3	5 (38.5)
Ib	1 (7.7)
IIb	1 (7.7)
IIIa	3 (23.1)
IIIb	
Pathologic downstaging	
Yes	13 (100)
Lymph node downstaging^a^	
Yes	6 (66.7)
No	3 (33.3)

a patients with N1-3 at baseline(number = 9).

### Surgery-Related Information

All patients underwent lobectomy and systemic lymph node dissection, with two (15.4%) patients undergoing open thoracotomy and 11 patients undergoing video-assisted thoracoscopic surgery (VATS, Table 3). None of the patients who received VATS experienced intraoperative conversion to open thoracotomy. All patients received complete resection. The median numbers of dissected lymph node station and number were 7 (IQR: 6–8) and 21 (IQR: 8–23), respectively. The median operative time was 115 min (IQR: 95–160). The median bleeding volume was 110 ml (IQR: 85–160). There were no perioperative deaths. The median postoperative hospital stay was 4 days (IQR: 3.3–6.2). The median chest placement time was 3 days (IQR: 2.6–4.4). Three (23.1%) patients had postoperative complications. One patient who underwent left upper lobe lobectomy experienced postoperative air leak for 5.5 days that improved with conservative treatment. The other two patients developed chylothorax on postoperative day 1 and day 2, respectively, and were cured by fatty food avoidance and octreotide administration.

**TABLE 3:** Surgery-related information.

Variables	
Extent of resection	
Lobectomy + ipsilateral lymphadenectomy	13 (100)
Surgical approach	
Open	2 (15.4)
VATS	11 (84.6)
Dissected nodal station, median (IQR)	7 (6–8)
Dissected nodal number, median (IQR)	21 (8–23)
Operative time, min, median (IQR)	115 (95–160)
Estimated blood loss, ml, median (IQR)	110 (85–160)
Thirty-day mortality	0
Ninety-day mortality	0
Postoperative hospital stay, days, median (IQR)	4 (3.3–6.2)
Duration of chest placement, days, median (IQR)	3 (2.6–4.4)
Postoperative complications	
Prolonged air leaks	1 (7.7)
Chylothorax	2 (15.4)
Surgical margin	
R0	13 (100)

### Toxicity Profile

Seven patients experienced grade 1 treatment-related adverse reactions during neoadjuvant therapy (Table 4). No adverse reactions of grade 2 or worse occurred. Pruritus (5/13, 38.5%) was the most common adverse reaction. 13 patients developed grade 1 treatment-related abnormal laboratory findings. Lymphocytopenia (5/13, 38.5%), anemia (3/13, 23.1%), and increased blood urea nitrogen (3/13, 23.1%) were the most common laboratory abnormalities. No patients developed grade 2 or worse laboratory abnormalities. No patients experienced drug withdrawal or surgical delays due to the adverse events.

**TABLE 4:** Toxicity profile.

	Grade 1
Any treatment-related adverse effects	7 (53.8)
Pruritus	5 (38.5)
Oral ulcers	2 (15.4)
Rash	1 (7.7)
Musculoskeletal pain	1 (7.7)
Acne	1 (7.7)
Any treatment-related abnormal laboratory findings	13 (100)
Anemia	3 (23.1)
Lymphocytopenia	5 (38.5)
Neutropenia	2 (15.4)
Thrombocytopenia	1 (7.7)
Hypoalbuminemia	2 (15.4)
Increased aminotransferases	2 (15.4)
Increased blood urea nitrogen	3 (23.1)

### Follow-Up

One patient was lost to follow-up. Regular postoperative follow-up was performed in the other 12 patients. The median follow-up was 9.5 months (range: 4.2–30.8 months). Up to March 2022, all 12 patients survived with no disease recurrence.

## Discussion

Targeted therapy has become the standard of care for EGFR-mutated metastatic lung cancer on the basis of improved PFS by comparison to chemotherapy (Rosell et al., 2012; Ramalingam et al., 2020). In view of its promising therapeutic potential in EGFR-mutated metastatic lung cancer, several scholars have started to use targeted therapy for early resectable EGFR-mutated lung cancer in the neoadjuvant setting Xiong et al., 2019; Zhang et al., 2021). Zhang et al. (2021) revealed that neoadjuvant gefitinib therapy achieved an ORR of 54.5% and an MPR of 24.2% in 33 patients with stage II-IIIa EGFR-mutated NSCLC, without severe toxicities observed (Zhang et al., 2021). Xiong et al. reported median PFS and OS in 19 patients with stage IIIA-N2 EGFR (+) NSCLC receiving neoadjuvant erlotinib were 11.2 and 51.6 months, respectively, with rates of ORR, R0 resection, and pathological downstaging being 42.1%, 68.4%, and 21.1%, respectively (Xiong et al., 2019). In addition, the incidence of AEs was 36.8%, with the most common being rash (26.3%) and the incidence of grade 3–4 AEs being 15.8%. Bao et al. (2021) retrospectively analyzed the clinical data of 42 patients with stage Ib-IIIc EGFR-mutated NSCLC treated with neoadjuvant first or second generation TKI targeted thrapy. Their results showed an ORR of 47.6% and an MPR of 23.8%, with grade 1/2 AEs of 82.9% and no grade 3/4 AEs (Bao et al., 2021).

Neoadjuvant chemotherapy is considered an acceptable therapy modality for resectable NSCLC (Brunelli et al., 2020). However, it remains unclear whether neoadjvant targeted therapy is superior to neoadjvant chemotherapy for resectable EGFR-mutated NSCLC. EMERGING-CTONG 1103 study compared the safety and efficacy of neoadjuvant erlotinib versus neoadjuvant chemotherapy in stage IIIA-N2 EGFR-mutated NSCLC (Zhong et al., 2019). Their results showed that the neoadjuvant erlotinib group was significantly better than the neoadjuvant chemotherapy group in terms of ORR rate (54.1% vs. 34.3%, *p* = 0.092), MPR rate (9.7% vs. 0) and median PFS (21.5 vs. 11.4 months, *p* < 0.001). However, there was no significant difference between the two groups regarding OS. The non-inferiority of neoadjuvant erlotinib regarding OS was likely associated with the small sample size and different adjuvant treatments in their study. Sun et al. (2020) performed a review of current prospective clinical trials of neoadjuvant first generation targeted therapies for resectable EGFR-mutated NSCLC (Sun et al., 2020). Although neoadjuvant TKI therapy provided satisfactory surgical outcomes and low drug toxicity, rates of pathologic downstaging and pCR were low. Whether neoadjuvant TKI therapy is superior to neoadjuvant chemotherapy and whether neoadjuvant TKI therapy, adjuvant TKI therapy, or the combination is more effective in improving the prognosis of patient with resectable EGFR-mutated NSCLC needs to be further validated in future phase 3 clinical trials.

Osimertinib, a third-generation TKI, has been approved in April 2018 as first-line treatment of EGFR-mutant advanced NSCLC or for the treatment of T790M-mediated resistance mutations following first-generation TKI therapy (Remon et al., 2018). However, evidence of osimertinib as neoadjuvant therapy for resectable non-small cell lung cancer are lacking. Chen et al. (2021) has reported the clinical efficacy of a stage IIIa NSCLC patient who received osimertinib as neoadjuvant therapy in combination with radiotherapy followed by surgery (Chen et al., 2021). A phase 3 NeoADAURA study has been initiated in 2020 to evaluate the safety and efficacy of neoadjuvant osimertinib alone or in combination with chemotherapy compared to chemotherapy alone for patients with stage II-IIIb EGFR-mutated NSCLC (Tsuboi et al., 2021). Recently, a phase II clinical trial evaluating the efficacy and safety of neoadjuvant osimertinib (80 mg QD for 6 weeks) for resectable EGFR-mutated lung adenocarcinoma (NEOS study) updated its findings at the 2022 ELCC meeting. This study showed neoadjuvant osimertinib therapy achieved an ORR of 71.1% and an R0 resection rate of 94%. Of the 28 patients evaluated pathologically, 11% achieved MPR, 4% achieved pCR, and 46% had ≥50% pathological remission. Moreover, neoadjuvant treatment did not significantly increase perioperative complications and was safe and well tolerated.

This case-series study aimed to assess the efficacy and safety of neoadjuvant osimertinib therapy for resectable EGFR-mutated NSCLC and our results showed similar clinical outcomes. In terms of radiologic response assessment, the ORR rate after neoadjuvant osimertinib therapy was 69.2%, which was similar to the results of NEOS study but significantly higher than the results (42%–54.5%) of neoadjuvant first generation TKIs studies (Rizvi et al., 2011; Zhang et al., 2021). This result was similar to the results of FLAURA study assessing osimertinib versus standard EGFR-TKIs in EGFR-mutated advanced NSCLC (Reungwetwattana et al., 2018). It seems that preoperative use of osimertinib is more likely to lead to radiologic remission compared with first-generation TKIs. Whether the improvement in ORR rate after neoadjuvant osimertinib therapy translates into a benefit in survival should be cautiously treated, considering RECIST criteria not being used as a valid surrogate measure of survival (Eisenhauer et al., 2009). In addition, our preliminary results showed that all patients received radical surgical treatment. In terms of pathological response assessment, neoadjuvant osimertinib therapy showed favorable outcomes, with all of the patients showing pathological downstaging and 66.7% of pN1-3 patients showing lymph node downstaging. However, no patients in this study achieved MPR or pCR. Similarly, only three (11%) patients obtained MPR in the NEOS study, and only one (4%) of them obtained pCR. Rate of MPR in thes two studies was clearly lower than the results of the above-mentioned neoadjuvant first-generation TKIs studies and the results of a series of neoadjuvant immunotherapy studies, including our previous study (Hu et al., 2021). We believe this phenomenon may be related to the small sample size of these preliminary studies. Furthermore, high degree of tumor heterogeneity and inevitably developed drug resistance to osimertinib may play a part (McCoach et al., 2016; Leonetti et al., 2019). Obviously, it is an area worth exploring in depth in the future. Previous studies have reported that neoadjuvant TKI therapy appears to be well tolerated (Sun et al., 2020). Neoadjuvant osimertinib therapy appeared not to increase the risk of surgery- and drug-related toxicity, in that no patients experienced delayed surgery or perioperative death, and the postoperative complication rate was only 23.1%, with a grade 1 AEs of only 53.8% observed. Although all patients had abnormal laboratory findings, no patient experienced a delay in surgery as a result.

There are several limitations needed to be acknowledged in the present study. First, this is a single-center retrospective study by design and therefore, there may be selection bias. Second, the sample size of this study was small and follow-up period was short. Third, heterogeneity regarding the duration of neoadjuvant therapy existed. Different drug exposure durations may have an impact on efficacy.

## Conclusion

Neoadjuvant osimertinib therapy seemed to be safe and feasible for resectable EGFR-mutated NSCLC. Future large prospective studies are warranted to confirm whether osimertinib as neoadjuvant therapy outperforms standard TKIs or chemotherapy for resectable EGFR-mutated NSCLC.

## Data Availability

The original contributions presented in the study are included in the article/Supplementary Material, further inquiries can be directed to the corresponding author.
